# Parkinson’s Disease Associated with GBA Gene Mutations: Molecular Aspects and Potential Treatment Approaches

**DOI:** 10.32607/actanaturae.11031

**Published:** 2021

**Authors:** K. A. Senkevich, A. E. Kopytova, T. S. Usenko, A. K. Emelyanov, S. N. Pchelina

**Affiliations:** Petersburg Nuclear Physics Institute named by B.P. Konstantinov of National Research Centre «Kurchatov Institute», Saint-Petersburg, 188300 Russia; First Pavlov State Medical University of St. Petersburg, Saint-Petersburg, 197022 Russia; Montreal Neurological Institute, McGill University, Montréal, QC, H3A 1A1, Canada; Institute of Experimental Medicine, St. Petersburg, 197376 Russia

**Keywords:** Parkinson’s disease, GBA, glucocerebrosidase, treatment

## Abstract

Parkinson’s disease (PD) is a multifactorial neurodegenerative disease. To date, genome-wide association studies have identified more than 70 loci associated with the risk of PD. Variants in the *GBA *gene encoding glucocerebrosidase are quite often found in PD patients in all populations across the world, which justifies intensive investigation of this gene. A number of biochemical features have been identified in patients with* GBA*-associated Parkinson’s disease (GBA-PD). In particular, these include decreased activity of glucocerebrosidase and accumulation of the glucosylceramide substrate. These features were the basis for putting forward a hypothesis about treatment of GBA-PD using new strategies aimed at restoring glucocerebrosidase activity and reducing the substrate concentration. This paper discusses the molecular and genetic mechanisms of GBA-PD pathogenesis and potential approaches to the treatment of this form of the disease.

## INTRODUCTION

Parkinson’s disease (PD) is a polyetiological neurodegenerative disease belonging to the class of synucleinopathies that also includes dementia with Lewy bodies (DLB) and multiple system atrophy (MSA) [1]. In synucleinopathies, neurodegeneration is caused by the accumulation and aggregation of the alpha-synuclein protein in the neuronal (PD, DLB) and glial (MSA) cells of the brain [1].

Pathomorphologically, PD is a neurodegenerative disease predominantly affecting the dopaminergic neurons of the substantia nigra and leading to the formation of protein aggregates in the cytoplasm of survived neurons; the so-called Lewy bodies, the main component of which is the alpha-synuclein protein [3, 4, 5].

PD is the most common synucleinopathy, with its incidence rate 1–3% in adults over 60 years of age [2]. Motor symptoms manifest after a loss of about 50–60% of the dopaminergic neurons of the substantia nigra [3, 4, 5]. However, the neurodegeneration process begins many years before the development of motor symptoms and can be characterized by a wide range of non-motor symptoms, such as constipation, olfactory disorders, depression, various sleep disorders (including rapid eye movement sleep behavior disorder (RBD)), etc. [6].

Despite the accepted term synucleinopathy, a number of genetically determined forms of PD have been recently found not to be associated with Lewy body formation. During autopsy, Lewy bodies were not found in more than 50% of patients with PD associated with *LRRK2 *gene mutations [7]. Aggregated alphasynuclein forms were also not found in the brain cells of patients with *PRKN *gene mutations [8]. Furthermore, Lewy bodies are absent in 8% of patients with sporadic PD (sPD) [9].

PD is known to be multifactorial in nature, and both genetic and environmental factors promote the development of the disease. To date, a number of genes associated with the development of PD have been identified [10]. The risk of PD is primarily associated with variants of the glucocerebrosidase (*GBA*) gene [11, 12, 13]. Mutations in the *GBA *gene are found in 5–20% of PD patients (depending on the population), with the highest rate being observed in Ashkenazi Jews [11]. Importantly, *GBA *gene mutations, despite their rather high rate in PD, have low penetrance. For example, 9–30% of carriers of *GBA *gene mutations at the age of 80 years and older develop clinical signs of the disease [14, 15, 16]. Of particular importance is the fact that *GBA* gene mutations are also associated with the development of other synucleinopathies, in particular DLB [17]. The data on the association of variants in the *GBA *gene with MSA remain controversial [18, 19, 20]. Recently, an association of *GBA *gene mutations with the development of RBD was established [21, 22]. More than 80% of patients with this disease develop PD or other synucleinopathies (DLB, MSA) [23].

This review discusses the molecular basis of GBA-PD pathogenesis and therapeutic approaches to the treatment of this form of the disease.

## GENETIC RELATIONSHIP BETWEEN PARKINSON’S DISEASE AND GAUCHER DISEASE

Gaucher disease (GD) is the most common lysosomal storage disease [24]. The development of this disease is associated with homozygous point mutations or heterozygous compound mutations in the *GBA *gene, which reduce the activity of glucocerebrosidase (GCase) [25, 26]. To date, more than 400 *GBA *gene mutations are known [27]. It should be noted that homozygous variants leading to a complete loss of GCase activity are lethal [28, 29]. Residual activity of the enzyme is required for the development of the body. Depending on the extent of a GCase activity decrease, both “favorable” and “unfavorable” variants of the gene are distinguished. The residual activity of GCase with “favorable” homozygous mutations (p.N370S, p.V394L, and p.R463C) accounts for 20–5% of the wild-type enzyme activity, while the residual activity of “unfavorable” variants is 5–0% (p.L444P) or absent (c.84dupG) [30, 31]. There are also polymorphic variants of the gene (p.E326K, p.T369M) associated with a decrease in GCase activity by up to 50% [30, 32], which do not lead to the development of GD in a homozygous state [33, 34].

There are three types of GD [35]; of these, type I with a favorable prognosis is the most common. At the end of the 20th century, there appeared a number of clinical case reports of patients with parkinsonism symptoms who were relatives of GD patients [36, 37, 38, 39].

In 2004, an association between *GBA *gene mutations and PD was first identified [40]. Later, this association was confirmed in a large-scale multicenter study [13]. The rate of *GBA *gene mutations in PD patients was found to vary in different populations [12, 41, 42, 43], prevailing among Ashkenazi Jews (up to 20%) [44]. Later, a 6- to 10-fold increase in the risk of PD in heterozygous carriers of *GBA *gene mutations was shown in many populations [12, 13, 43]. The carriage of p.E326K and p.T369M variants was found to increase the risk of PD 1.5- to 2-fold [12, 45, 46]. In this case, the risk of PD does not depend on the homozygous/heterozygous carrier status of *GBA *gene mutations [16]. However, the PD phenotype and the age of disease onset were shown to be associated with the type of mutation [11, 47, 48].

## PHENOTYPIC FEATURES OF GBA-PD PATIENTS

GBA-PD patients are characterized by a special phenotype: the disease begins earlier than in sporadic PD (sPD) [48]; non-motor symptoms, including cognitive deficit, are more pronounced, and the rate of disease progression is higher than in sPD [49, 50, 51, 52, 53, 54]. Also, GBA-PD patients are characterized by more frequent hallucinations and a higher risk of depression and anxiety [47, 53, 55, 56, 57]. In this case, cognitive impairments and mental symptoms are more typical of carriers of “unfavorable” mutations (p.L444P, c.84dupG, 370Rec) than carriers of more “favorable” alleles (p.N370S) [47]. Interestingly, cognitive impairments also prevail in carriers of gene variants associated with a slight increase in the risk of PD (p.E326K, p.T369M) in comparison with sPD patients [58].

## FUNCTION OF GCase IN HEALTH AND DISEASE

**Fig. 1:**
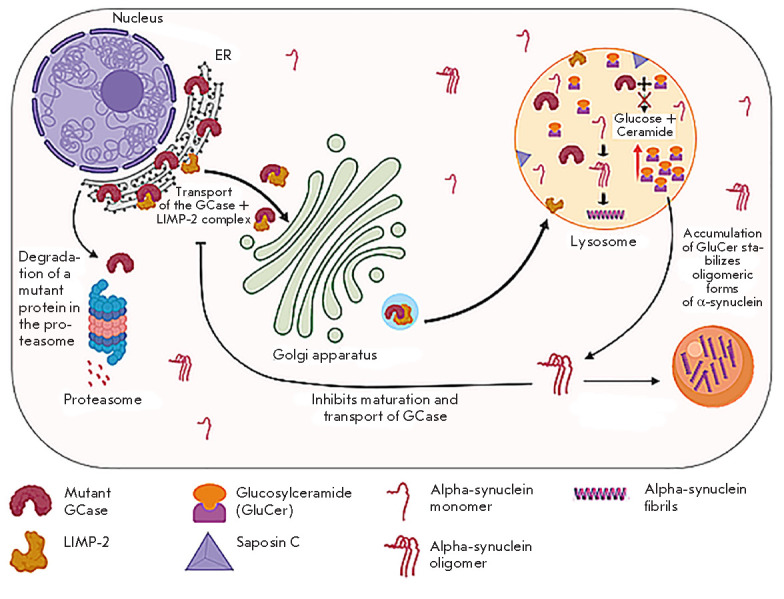
Metabolism of GCase and possible interaction with alpha-synuclein

The *GBA *gene encodes the lysosomal enzyme GCase that cleaves glucosylceramide (GlcCer) into glucose and ceramide. GCase is a membrane-bound protein with five glycosylation sites [27, 59]. A decrease in the enzyme activity is accompanied by lysosomal accumulation of GlcCer and the lysosphingolipid glucosylsphingosine (GlcSph) formed during deacetylation of GlcCer. Accumulation of these substances in lysosomes of GD patients leads to the formation of phenotypically altered macrophages, the so-called Gaucher cells. Accumulation of Gaucher cells in various organs and tissues leads to the development of GD symptoms (changes in bones, hepatosplenomegaly, anemia) [60]. Synthesis of the protein encoded by a mutant *GBA *gene in the endoplasmic reticulum (ER) is accompanied by misfolding as well as changes in the native conformation of the enzyme and its transport into lysosomes (*Fig. 1*). After maturation in the ER, the protein binds to the lysosomal integral membrane protein 2 (LIMP-2). The LIMP-2 protein encoded by the *SCARB2 *gene provides GCase transport from the ER to lysosomes, where the proteins dissociate under acidic conditions [61]. Altered* LIMP-2 *expression in PD model mice was shown to lead to a decrease in GCase activity and damage to dopaminergic neurons, mediated by the accumulation of alpha-synuclein [62].

Transport of the GCase–LIMP-2 complex into the lysosome is facilitated by various proteins. In particular, these include the heat shock protein HSP70 with progranulin, as a cochaperone [63]. Furthermore, progranulin was shown to modulate GCase activity [64, 65]. Interestingly, the locus of the *GRN *gene, which encodes progranulin, and variants in the *SCARB2 *gene are associated with the development of PD [66, 67, 68].

Co-factor proteins are required for functional activity of GCase. An acidic environment in lysosomes is favorable for the functioning of GCase; however, the saposin C protein is required to increase the catalytic activity of the enzyme [69]. The lysosomal protein saposin C provides maximum GCase activity and prevents proteolysis of the enzyme [70]. Saposin C is supposed to bind the protein with GlcCer and directs the substrate to the enzyme active center [69]. Saposin C is one of three proteins encoded by the *PSAP *gene. Rare mutations in this gene lead to the development of GD [71]. However, no association between variants in the *PSAP* gene and PD has been found [72].

The pathogenesis of GBA-PD is unclear. A decrease in GCase activity could cause lysosomal dysfunction and, subsequently, a reduction in alpha-synuclein degradation. Studies, including *in vitro*, in animal models and *post mortem *have revealed a number of features of the interaction between GCase and alpha-synuclein, which suggest a molecular basis of GBA-PD pathogenesis. A physical interaction between GCase and alpha-synuclein was found in an acidic environment* in vitro *[73, 74]. As mentioned, GCase is a membranebound protein. The interaction between GCase and alpha-synuclein can lead to the formation of a membrane GCase–alpha-synuclein complex. This structure is supposed to increase the efficiency of alphasynuclein cleavage by proteases [59]. Also, impaired degradation of alpha-synuclein in lysosomes can lead to a decrease in GCase activity [75, 76] and an increase in alpha-synuclein aggregation [75, 76]. In this case, lipids of the lysosomal membrane and sphingolipids, in particular, can affect alpha-synuclein aggregation [77, 78]. Furthermore, *in vitro *and *in vivo *studies have shown an interaction between GlcCer and GlcSph sphingolipids and alpha-synuclein, which can lead to the accumulation of neurotoxic forms of the protein, due to its oligomerization [75, 79, 80]. Experiments on a neuronal cell culture have also demonstrated that sphingolipids promote alpha-synuclein aggregation [81]. Accordingly, a decrease in the synthesis of glucosylceramide leads to a reduction in the alpha-synuclein concentration [82]. Recently, an inverse correlation was uncovered between the GCase protein level and the ratio of alpha-synuclein phosphorylated at Ser129 to total alpha-synuclein [83]. Modeling of potential pathogenic pathways suggested that the effect of GCase dysfunction on an increase in the phosphorylated alpha-synuclein level is partly due to an increase in the glucosylsphingosine level in the substantia nigra [83].

While a decrease in blood GCase activity and accumulation of lysosphinglipids are considered GD biomarkers [35], no changes in these parameters in heterozygous carriers of *GBA *gene mutations could be detected for a long time. By using modern methods for determining GCase activity and metabolite concentrations (liquid chromatography with tandem mass spectrometry), we and other authors have uncovered a decrease in blood GCase activity in GBA-PD patients [32, 84]. An increase in the blood lysosphingolipid concentration was shown in GBA-PD [85, 86]. A decrease in GCase activity was also established in blood cells of sPD patients [32]; however, these data could not be confirmed in a number of studies [84, 87, 88]. A decrease in GCase activity in the cerebrospinal fluid and substantia nigra of sPD patients was also shown [89, 90, 91]. But it should be noted that GCase activity decreases with age [92].

Therefore, according to the most circulated hypothesis of the PD developmen mechanism in carriers of *GBA *gene mutations, accumulation of GlcCer and GlcSph is related to a decrease in the enzymatic activity of GCase (loss of function), which leads to impaired autophagy and oligomerization of alpha-synuclein [75].

Earlier, we identified an increase in the concentration of oligomeric forms of alpha-synuclein in the blood plasma of patients with both GD and GBA-PD [84, 93, 94]. Also, accumulation of alpha-synuclein and a decrease in GCase activity were found in various parts of the brain in sPD [90]. Accumulation of sphingolipids and alpha-synuclein aggregates in the brain and their co-localization were demonstrated in animal models of parkinsonism [79]. An inverse correlation among GCase activity, cognitive dysfunction, and motor deficits was found in model animals [82]. Therefore, a slight, but long-term decrease in the enzymatic activity of GCase may be a trigger for the accumulation of alpha-synuclein. As already mentioned, GBA-PD patients have a special clinical phenotype [49, 50, 51, 53, 56, 57] with a predominance of cognitive impairment, anxiety, and depression [53, 56, 95]. A similar phenotype is characteristic of patients with mutations and multiplications of the *SNCA *gene encoding alpha-synuclein [96, 97]. Probably, GBA-PD and *SNCA*-associated PD develop in a similar pathogenic pathway and have a similar phenotypic picture.

However, there exist data inconsistent with the hypothesis discussed above. For example, autopsy material of the substantia nigra from GBA-PD patients was characterized by a decrease in GCase activity [89, 98, 99] and no increase in the concentration of sphingolipids [100]. According to an alternative hypothesis (gain of function), due to mutations, GCase acquires a toxic function and disrupts the ER and protein transport in the cell [101].

There exist also data on the impact of inflammation on alpha-synuclein aggregation and PD development [102]. Alpha-synuclein was shown to be capable of directly provoking an inflammatory response [103, 104]. We and other authors have found that the blood concentration of cytokines in GBA-PD patients is increased compared to that in sPD [105, 106].

## POTENTIAL THERAPEUTIC APPROACHES FOR GBA-PD

To date, PD therapy remains completely symptomatic and fails to slow down the rate of neuron loss in the brain. Today, there are no drugs capable of preventing or slowing down the development of the disease. Levodopa, proposed in 1961, remains the gold standard of treatment [107]. The search for drugs or compounds that have a therapeutic or neuroprotective effect is considered a priority in PD research.

The known molecular features of GBA-PD were used to hypothesize a possible preventive and therapeutic effect of drugs aimed at increasing GCase activity and reducing the concentration of sphingolipids. Clinical trials of several drugs are currently under way (*Table*). It should be noted that a prerequisite for the use of these drugs in the treatment of PD is their ability to pass through the blood-brain barrier (BBB).

**Table:** Clinical trials of drugs targeting GBA-PD

Drug	Pharmacological group	Mechanism	Phase
Ambroxol	Pharmacological chaperone	Activation of GCase	II
Venglustat (GZ/SAR402671)	Substrate reduction therapy	A decrease in the substrate concentration (inhibition of glucosylceramide synthase)	II
LTI-291	Pharmacological chaperone	Allosteric activator of GCase	Ib

The known molecular features of GBA-PD were used to hypothesize a possible preventive and therapeutic effect of drugs aimed at increasing GCase activity and reducing the concentration of sphingolipids. Clinical trials of several drugs are currently under way (*Table*). It should be noted that a prerequisite for the use of these drugs in the treatment of PD is their ability to pass through the blood-brain barrier (BBB).

Currently, treatment of GD involves enzyme replacement therapy (ERT) and substrate reduction therapy [108, 109]. In the former case, intravenous administration of a recombinant GCase enzyme is employed [109]. ERT drugs are successfully used in type I GD. However, these drugs do not pass through the BBB; so, they do not exhibit a therapeutic effect on neurological symptoms in patients with type II and type III GD and cannot be effective in PD.

**Fig. 2:**
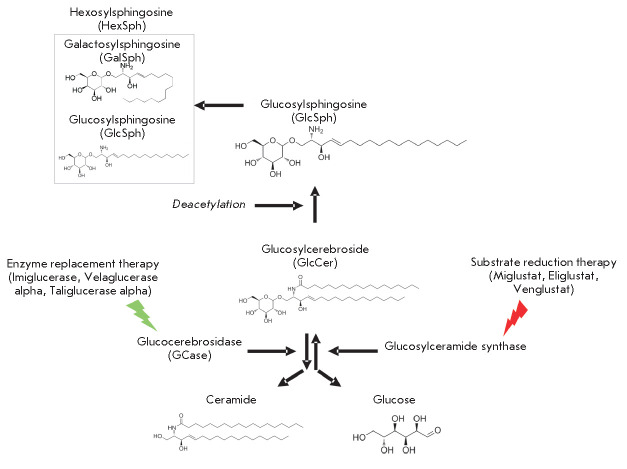
Drugs for the treatment of Gaucher disease

Substrate reduction therapy could potentially relieve the symptoms of PD. Currently, miglustat and eliglustat are used for the treatment of GD [110, 111] (*Fig. 2*). The action of these drugs is based on a selective inhibition of GlcCer biosynthesis through the inhibition of glucosylceramide synthase, which decreases the GCase substrate level [108, 109]. It should be noted that miglustat, despite its ability to penetrate the BBB, was ineffective in neuropathic forms of GD [112]. In this case, the development of therapeutic agents of this class passing more efficiently through the BBB should modify the clinical course of neuropathic forms of GD and GBA-PD [82, 113]. The first clinical trial of a drug in this group is currently underway in GBA-PD patients. Phase I clinical trials have shown that venglustat can penetrate into the central nervous system; phase II trials are underway (https://www.clinicaltrials.gov/ ct2/show/study/NCT02906020).

In the case of GBA-PD, the most promising area is the search for small chemical compounds, pharmacological chaperones, which bind to enzymes, facilitating their folding and transport to organelles. This strategy is considered as a potential approach to increasing the enzymatic activity of GCase, because most *GBA *gene mutations result in amino acid substitutions outside the enzyme active site, which disrupt GCase activity, affecting the maturation of this protein. The action mechanism of pharmacological chaperones involves their binding to GCase, which promotes the correct assembly of the enzyme in the ER and its transport to lysosomes, where dissociation of a substance and the GCase enzyme occurs under low pH conditions [114].

One of these substances is ambroxol hydrochloride (ambroxol), which is registered as a drug that reduces mucus hypersecretion in the respiratory tract and is used in the treatment of the hyaline membrane disease in newborns. The modulating effect of ambroxol on GCase was reported in 2009 [115]. The effectiveness of ambroxol in restoring the enzymatic activity of GCase has been demonstrated both in cell lines and in animal models of parkinsonism. Ambroxol has been repeatedly tested *in vitro *[115, 116, 117, 118, 119] and *in vivo *[120, 121, 122, 123].

Our team and other authors have shown that a primary culture of macrophages derived from the pe- ripheral blood monocytes of GBA-PD and GD patients can be used for personalized screening and assessment of the effectiveness of pharmacological chaperones [124, 125]. Peripheral blood macrophages from GD and GBA-PD patients, which were cultured in the presence of ambroxol, demonstrated an increase in GCase activity and a decrease in the concentration of lysosphingolipids [124, 125, 126]. Recent data have demonstrated that the effects of ambroxol can depend on the type of *GBA* gene mutations. Ambroxol was less effective in a line of fibroblasts from GD patients with “unfavorable” *GBA* gene mutations (e.g., L444P/L444P or D409H/L444P) than in GD patients with the N370S/N370S mutation [124]. The ability of ambroxol to pass through the BBB and increase GCase activity, and reduce alphasynuclein aggregation, was shown in PD animal models [127].

The first clinical trial of ambroxol for the treatment of GBA-PD was recently completed. This open-label, non-randomized, non-controlled study included 18 PD patients (8 GBA-PD, 10 PD) who received oral ambroxol [119]. The drug proved safe and had the ability to pass through the BBB. The patients had improved clinical symptoms; however, it should be noted that a small sample of patients and the absence of a placebo control group complicate any interpretation of the results [119]. Currently, the effectiveness of ambroxol in the treatment of PD with dementia is under study [128].

Another pharmacological chaperone of GCase is the iminosugar isophagomine [129]. *In vitro *and *in vivo* studies have shown the effectiveness of isophagomine in restoring mutant GCase activity, reducing the level of substrates, and decreasing the rate of neurodegeneration [114, 130, 131].

Clinical studies of isophagomine for the treatment of GD have revealed the safety and satisfactory tolerability of the drug. However, the clinical effect was minimal, and the third phase of the studies was not performed (https://ir.amicusrx.com/news-releases/ news-release-details/amicustherapeutics-announcespreliminary- results-phase2-study).

Also, a clinical study of another GCase molecular chaperone (LTI-291 (LTI/Allegran)) has been registered. This study, assessing the effectiveness of the drug in the treatment of GBA-PD, is undergoing phase 1b testing (https://www.trialregister.nl/trial/7061) (*Table*).

We have constructed an *in silico *model of mutant GCase with allowance for the enzyme glycosylation sites [132]. Using molecular docking methods, we have searched for possible modifications of allosteric pharmacological chaperones of GCase which increase their binding to the enzyme and, as a consequence, their effectiveness in restoring the enzymatic activity of GCase (unpublished data).

## CONCLUSION

An investigation of the pathogenic basis of GBA-PD has identified new therapeutic targets in a short time. The challenge is the expansion of a GBA-PD patient cohort for clinical trials. Of great importance is the screening of *GBA *gene mutations in PD patients for their potential enrollment in clinical trials. The scale of research to identify new GCase activators and the increasing number of compounds approved for clinical trials suggest that GBA-PD may become the first form of parkinsonism for which new therapeutic approaches are developed.
